# Role for the Ventral Posterior Medial/Posterior Lateral Thalamus and Anterior Cingulate Cortex in Affective/Motivation Pain Induced by Varicella Zoster Virus

**DOI:** 10.3389/fnint.2017.00027

**Published:** 2017-10-16

**Authors:** Phillip R. Kramer, Jennifer Strand, Crystal Stinson, Larry L. Bellinger, Paul R. Kinchington, Michael B. Yee, Mikhail Umorin, Yuan B. Peng

**Affiliations:** ^1^Department of Biomedical Sciences, Texas A&M University College of Dentistry, Dallas, TX, United States; ^2^Department of Psychology, University of Texas at Arlington, Arlington, TX, United States; ^3^Department of Ophthalmology and Molecular Microbiology and Genetics, Eye and Ear Institute, University of Pittsburgh, Pittsburgh, PA, United States

**Keywords:** ventral posterolateral nucleus, ventral posteromedial nucleus, anterior cingulate cortex, orofacial pain, varicella zoster virus, shingles, post-herpetic neuralgia

## Abstract

Varicella zoster virus (VZV) infects the face and can result in chronic, debilitating pain. The mechanism for this pain is unknown and current treatment is often not effective, thus investigations into the pain pathway become vital. Pain itself is multidimensional, consisting of sensory and affective experiences. One of the primary brain substrates for transmitting sensory signals in the face is the ventral posterior medial/posterior lateral thalamus (VPM/VPL). In addition, the anterior cingulate cortex (ACC) has been shown to be vital in the affective experience of pain, so investigating both of these areas in freely behaving animals was completed to address the role of the brain in VZV-induced pain. Our lab has developed a place escape avoidance paradigm (PEAP) to measure VZV-induced affective pain in the orofacial region of the rat. Using this assay as a measure of the affective pain experience a significant response was observed after VZV injection into the whisker pad and after VZV infusion into the trigeminal ganglion. Local field potentials (LFPs) are the summed electrical current from a group of neurons. LFP in both the VPM/VPL and ACC was attenuated in VZV injected rats after inhibition of neuronal activity. This inhibition of VPM/VPL neurons was accomplished using a designer receptor exclusively activated by a designer drug (DREADD). Immunostaining showed that cells within the VPM/VPL expressed thalamic glutamatergic vesicle transporter-2, NeuN and DREADD suggesting inhibition occurred primarily in excitable neurons. From these results we conclude: (1) that VZV associated pain does not involve a mechanism exclusive to the peripheral nerve terminals, and (2) can be controlled, in part, by excitatory neurons within the VPM/VPL that potentially modulate the affective experience by altering activity in the ACC.

## Introduction

Varicella zoster virus (VZV) causes chickenpox and shingles resulting in acute and chronic pain. VZV infects cutaneous innervating axons, then transports to the neuronal cell body to undergo replication and later viral latency. During the latency period the VZV DNA remains in sensory ganglia, but there is little to no virus production. In a third of VZV infected individuals VZV reactivates, typically after age 50, to cause herpes zoster (HZ; i.e., shingles). HZ is debilitating and painful, with up to 90% of patients seeking pain alleviating medication (Johnson et al., 2010). The current live shingles vaccine will only reduce the incidence of HZ from one in three people to one in six (Harpaz et al., 2008) and there will still be 1 million people that develop HZ each year (in US). Thus, while the widespread use of the chickenpox vaccine has greatly reduced chickenpox incidence, most adults over 35 have not had the vaccine and harbor wild type VZV with the potential to reactivate.

The trigeminal ganglion contains the greatest latent viral burden and about 80% of trigeminal ganglion neurons harbor detectable VZV DNA (Pevenstein et al., 1999). As such, orofacial zoster and post-herpetic neuralgia is common in the facial region, with greater than 13% of HZ cases having facial involvement (Ragozzino et al., 1982; Pavan-Langston, 1995; Pevenstein et al., 1999). This includes HZ opthalmicus (HZO), in which there is not only head and facial pain, but also ocular pain. Approximately 50,000 individuals in the US have HZ related vision problems every year (Pavan-Langston, 2000) thus, the need to study pain related to VZV infection of the orofacial region is needed.

The mechanisms underlying VZV-induced chronic pain are unclear, but it appears there is no need for ongoing VZV replication. Most patients given antiviral acyclovir (which inhibits viral DNA replication) show no improvement (Surman et al., 1990; Hempenstall et al., 2005). Like that seen in humans, administration of antiviral agents to a rat does not affect the paw affective pain after VZV infection (Dalziel et al., 2004). Treatment of VZV infected animals with antivirals, opioids or NSAIDS is not highly effective, mirroring results in humans with pain after VZV infection (Rowbotham and Fields, 1996; Dalziel et al., 2004; Garry et al., 2005; Hasnie et al., 2007). Both sodium channel blockers and gabapentin can effectively treat VZV-induced pain in animals and humans (Garry et al., 2005; Hasnie et al., 2007; Rullán et al., 2017; Stinson et al., 2017). Thus, the rat model reflects aspects of the human disease and would provide information as to the mechanism by which VZV induces pain.

VZV injection of the whisker pad resulted in neurites to retract within the tissue (Stinson et al., 2017). This result is consistent with humans that have VZV-induced neuralgia (Guedon et al., 2015). Knowing that peripheral neurites show damage it was necessary to determine if the mechanism for VZV pain had an exclusive origin in the peripheral nerve. In the current article, we address this question by measuring affective pain responses following infusion of VZV directly into the trigeminal ganglion and thus circumventing peripheral nerve terminal infection.

Previous work in our laboratory suggested that a central mechanism was involved, specifically that GABA within the ventral posterior medial/posterior lateral thalamus (VPM/VPL) modulated VZV-induced nociception (Kramer et al., 2017). The question remained as to what role specific cellular populations within the lateral thalamus had in VZV-induced pain. In addition, a functional connection between the anterior cingulate cortex (ACC) and VPM/VPL had been observed previously (Wang et al., 2007) and it was unclear if there was any association between the ACC and the VZV-induced response.

To address the role of central processing in VZV-induced pain and to determine if the mechanism for VZV pain is exclusive to the peripheral nerves a place escape avoidance paradigm (PEAP) was used to measure VZV-induced affective pain in the orofacial region of the rat. Pain measurements were completed after VZV injection of the trigeminal ganglion. VPV Local field potentials (LFPs) were measured in the VPM/VPL and ACC of awake rats after modulating neuronal activity in the VPM/VPL with a designer receptor exclusively activated by a designer drug (DREADD). Immunostaining was completed to identify the cell type controlled by the DREADD constructs. These studies were completed to determine a role for the central nervous system in VZV-induced affective pain in an awake animal.

## Materials and Methods

### Animal Husbandry

This study was approved by the Texas A&M University College of Dentistry Institutional Animal Care and Use Committee. Male (280–300 g) Sprague-Dawley rats from Envigo (Indianapolis, IN, USA) were kept on a 14:10 light/dark cycle. The rats were given food and water *ad libitum*. After a 4 day acclimation period experiments were carried out in accordance with the NIH regulations on animal use.

### Experiments

Three experiments were completed in total (Table 1). In Experiment 1 the left trigeminal ganglion was infused with either 2–3 × 10^4^ pfu of VZV (MeWo cells containing VZV, the parent Oka strain) or an equivalent amount of control (MeWo) cells; there were 11 animals in the control group and 17 animals in the VZV treatment group. Technical note: VZV is unstable and must be administered within MeWo cells that produce the virus particles. A behavioral affective pain assay was then completed for 2 weeks.

**Table 1:** Experimental outline for testing VZV pain.

Experiment	Question	Experimental groups		Experimental tests		Number of animals	
Experiment #1: Inject trigeminal ganglia with VZV	Does VZV induce pain without damage to the terminals of the sensory neurons?	Control (TG infusion)		Affective pain		11	
		VZV (TG infusion)		Affective pain		17	
Experiment #2: VPM/VPL infusion with DREADD and whisker pad injection with VZV	Can neuronal inhibition in the VPM/VPL reduce orofacial affective pain?	Whisker pad injection	IP injection			4–5	
		Control	Vehicle	Affective pain		4–5	
		Control	CNO	Affective pain		4–5	
		VZV	Vehicle	Affective pain		4–5	
		VZV	CNO*	Affective pain		4–5	
Experiment #3: VPM/VPL infusion with DREADD and whisker pad injection with VZV	Can neuronal inhibition in the VPM/VPL reduce neuronal activity in the VPM/VPL and alter activity in the ACC?	Whisker pad injection	IP injection				
		Control	Vehicle	LFP	ICC	7	3
		Control	CNO	LFP	ICC	7	3
		VZV	Vehicle	LFP	ICC	7	4
		VZV	CNO	LFP	ICC	7	4

Abbreviations: VZV, varicella zoster virus; CNO, clozapine-n-oxide; LFP, local field potentials; IP, intraperitoneal injection; ICC, immunocytochemistry. *All animals in Experiment #2 and Experiment #3 had designer receptors exclusively activated by designer drug (DREADD) infused into the ventral posterior medial/posterior lateral thalamus but there was a VZV injected group that received CNO and did not have the DREADD construct infused, shown in Supplementary Figure S3.

In Experiment 2, the VPM/VPL of 18 rats was infused with a neuronal silencer adeno-associated virus (AAV8-DREADD) and then a week later the whisker pad was injected with 100,000 pfu of VZV or control (4–5 rats per treatment group). Behavioral affective pain assays were completed, with and without activation of the DREADD, for 3 weeks in these animals.

In Experiment 3, the VPM/VPL of 28 rats (7 rats per treatment group) was infused with AAV8 that resulted in the transduction of the neuronal silencer DREADD construct. Recording electrodes were implanted in the VPM/VPL and ACC. A week later the whisker pad was injected with 100,000 pfu of VZV. LFP recording in awake animals was completed once a week for 3 weeks, after which time the rats were sacrificed and tissues collected for molecular studies. Three rats were randomly chosen out of the control groups and four rats out of the VZV treated groups for perfusion and immunostaining, the remaining tissues were fresh frozen for future molecular analysis. The recording backpack restricts movement and significantly effects behavior (data not shown), thus behavioral measurements were not completed in Experiment 3 but were completed in Experiment 2 where the animals were treated the same but did not have electrophysiological recording. Experiments using VZV were completed under using Biosafety level II approved protocols.

### Experiment 1—Trigeminal Ganglion VZV Infusions

The rats were anesthetized with 2% isoflurane and an air flow of 2 liter per minute. Stereotaxic (David Kopf Instruments, Tujunga, CA, USA, Model 1460-61) infusions of VZV were performed (Hamilton #7002KH Neuros syringe, Reno, NV, USA) at coordinates AP = 1.5 mm from Bregma, 2.0 mm from midline and 11.2 mm from the top of the skull. These coordinates correspond to neurons of the trigeminal ganglion that innervate the V1 and V2 branch of the trigeminal nerve (Leiser and Moxon, 2006). The V1 and V2 branches innervate the eye and upper face (Leiser and Moxon, 2006). The trigeminal nerve is the most common cranial nerve involved in zoster of the face followed by the glossopharyngeal and hypoglossal nerves (Kaur et al., 2016). Regions of the face innervated by the V1 branch of the trigeminal ganglion are the most common in presenting zoster and regions innervated by V2 and V3 are less commonly involved in HZ of the face (Millar and Troulis, 1994) thus, we targeted the more commonly infected neurons of the trigeminal ganglion in these studies. The left trigeminal ganglion was infused with 0.5 ml of MeWo cells containing VZV (2–3 × 10^4^ pfu/ganglion) or MeWo cells (control). A Stoelting (Woodale, IL, USA) stereotaxic syringe pump system was used to infuse at a rate of 0.05 microliters per minute. The infusion needle was left in place 5 min before removing.

In a pilot experiment 0.5 microliter of India ink was infused as described above into the trigeminal ganglia to verify placement. Animals were sacrificed and perfused immediately after injection. Trigeminal ganglion tissue was sectioned and stained with hematoxylin and eosin. Sections were imaged using a Nikon fluorescent microscope and NIS-Elements imaging software and a Photometrics CoolSnap K4 CCD camera (Roper Scientific, Inc, Duluth, GA, USA).

### Experiment 2—Thalamic Neuronal DREADD Infusion

The rats were anesthetized with 2% isoflurane and an air flow of 2 liter per minute. Stereotaxic infusion into the right VPM/VPL with AAV8 was performed at coordinates 3.6 mm posterior of Bregma, 3.0 mm from midline at a depth of 6.0 mm from the top of the skull. The pump infused 0.250 μl of 2–8 × 10^12^ pfu/ml AAV8 at a rate of 20 nanoliters per minute. After infusion the needle was left in place for 5 min and then removed. Using these coordinates and injection procedure the modified acetylcholine Gi protein-coupled receptor was expressed primarily in the VPM, VPL with a smaller amount of expression in the reticular (Rt) thalamic nuclei, as well as, the zona incerta (ZI) of Sprague-Dawley rats (Kramer et al., 2017).

The AAV8 virus expressed a neuronal silencing construct hSyn-hM4D(Gi)-mCherry (Gene Therapy Center Vector Core, University of North Carolina at Chapel Hill) or vehicle. The hM4D(Gi) gene was an engineered acetylcholine Gi-protein coupled receptor that inhibits neuronal firing when bound by clozapine-n-oxide (CNO; Pei et al., 2008). Expression of the receptor was driven by the neuronal synapsin-1 promoter (Syn), which drives expression in most neurons.

Upon CNO binding the engineered acetylcholine Gi receptor stimulates calcium release, ERK1/2 activation, inhibits forskolin-induced cAMP formation and potentially GIRK activation, thereby causing hyperpolarization and inhibition of basal action potential firing (Rogan and Roth, 2011).

The rats received an intraperitoneal injection of 1 mg/kg CNO dissolved in 0.9% saline or an injection of 0.9% saline in a 0.5 ml volume 30 min before testing. The rats were tested once a week for 3 weeks.

### Experiment 3—Neuron Silencer Infusion and Recording Electrode Placement

Immediately after infusion of AAV8 into the VPM/VPL, two stainless steel bipolar twisted recording electrodes (MS303-1-B-SPC Elect SS 2C TW, Plastics One, Roanoake, VA, USA) were implanted into the right VPM/VPL or right ACC. Thalamic coordinates were 3.6 mm posterior of Bregma, 3.0 mm from midline at a depth of 6.0 mm. The second recording electrode was placed in the ACC at coordinates 1.0 mm posterior from Bregma, 0.7 mm lateral of midline at a depth 3.1 mm at a 26° angle. The recording electrodes were cemented with acrylic to jewelers screws attached to the skull. Histology was performed after sacrifice to verify that electrode placement was correct.

The rats received an intraperitoneal injection of 1 mg/kg CNO dissolved in 0.9% saline or an injection of 0.9% saline in a 0.5 ml volume 30 min before testing. The rats were tested once a week for 3 weeks.

### Place Escape/Avoidance Paradigm Affective Pain Testing

Because the magnitude of the behavioral response in the PEAP test has been shown to be greater than the thermal and mechanical response after VZV injection, all testing in this study was completed using the PEAP test (Stinson et al., 2017). Spontaneous behavioral responses were not significantly affected by VZV injection (Supplementary Figure S1).

In Experiments 1 and 2 rats were anesthetized briefly with 2% isoflurane using a 2 liter per minute flow of air and the left whisker pad was injected with 100 μl of MeWo cells infected with VZV or control MeWo cells lacking virus. Whisker pad injections were completed 1 week after thalamic infusion and electrode surgeries. The rats were ambulatory 5 min after whisker pad injection.

Seven days following whisker pad injection rats were evaluated for affective pain by individuals blinded to the groups using the PEAP test, as detailed by the Fuchs lab (LaBuda and Fuchs, 2000). In this test rats are placed in a 30 cm × 30 cm × 30 cm acrylic box where half the box is covered in black cloth. The PEAP test is based on the assumption that an aversive stimulus results in an escape and/or avoidance behavior (LaBuda and Fuchs, 2000). Rodents, being nocturnal, prefer the dark side of the chamber.

Animals in the chamber were then poked with a 60 g Von Frey filament every 15 s, applying stimulus to the injected side when rats are on the dark side of the chamber, and to the un-injected side when on the light side of the chamber. Stimuli were applied to the region below the eye and caudal to the whisker pad, a region innervated by the second branch of the trigeminal ganglion (Leiser and Moxon, 2006). The time spent on the dark side of the box was recorded during the total testing time of 30 min. The 30 min test was performed weekly. Data is reported as the time spent or the percent of time spent on the light side during the 30 min of testing.

The rats in Experiment 1 were tested once a week for 2 weeks, while the rats in Experiment 2 were tested weekly for 3 weeks.

### Recording VPM/VPL Electrical Activity

LFP records the summation of electrical currents (Zheng et al., 2012) primarily contributed through EPSPs and IPSPs, but also contributed by low frequency activity such as non-synaptic calcium spikes, glial cell fluctuations and other subthreshold membrane oscillations (Berens et al., 2008). The use of awake and behaving animals for LFP recording is important in capturing neuronal activity accurately because anesthesia can alter the power at multiple frequency levels (Sellers et al., 2015).

In Experiment 3, LFP recordings were made using a customized wireless recording module (Zuo et al., 2012) attached to a recording electrode implanted directly into the VPM/VPL and ACC. LFP was recorded using a low pass filter of 100 Hz. Standard techniques of applying a fast Fournier transform (FFT) were used (Masimore et al., 2004). The time resolution was 0.8192 s and the frequency resolution was 1.221 Hz.

Before recording the rats were briefly (5 min) anesthetized with 2% isoflurane and fitted with a backpack containing the LFP customized wireless recording module (Zuo et al., 2012) and then the module was connected to the recording electrode. After waking from anesthesia a 10 min LFP “Baseline” was recorded. Recording was completed for another 30 min after intraperitoneally injecting a 1 mg/kg dose of CNO in 0.9% saline or injecting no CNO (0.9% saline) in a 0.5 ml volume (Armbruster et al., 2007). This showed CNO alone did not affect the recordings. Next, rats were recorded for 30 min while poking the left whisker pad every 15 s in induce nociception. The total recording time of the experiment was 70 min. Testing was repeated once a week for 3 weeks.

Signals from the electrode were amplified and changed from volts to digital form by an Analog-to-Digital Converter (C8051F920, Silicon Laboratories, Austin, TX, USA) within the recording microcontroller module. Signals were transmitted by a receiver on a USB dongle to a laptop using customized software. The signals were saved as data in a text file and imported as a raw waveform at a sampling rate of 4096 Hz (CED Spike2 V7 software, Cambridge, UK).

### Immunofluorescent Staining

In Experiment 3, the animals were anesthetized with 100 mg/kg ketamine and 10 mg/kg xylazine the animals were first perfused with 9% sucrose and then with 4% paraformaldehyde in 1× PBS, pH 7.4. Fixed tissues were stored in 25% sucrose, frozen, cryo-sectioned and the 20 μm sections placed on Histobond slides (VWR international, Radnor, PA, USA).

The tissue was then blocked with a PBS solution containing 5% normal goat serum and 0.3% Triton-X 100 for 2 h at room temperature. The slides were incubated in primary antibody or the dilution buffer overnight at 4°C. Primary antibodies consisted of the mouse monoclonal NeuN antibody at a 1:250 dilution (Millipore, Billerica, MA, USA), or the mouse monoclonal vesicular glutamate transporter 2 (VGLUT2) antibody (ab79157, Abcam, Cambridge, UK) at a 1:150 dilution. Primary antibodies were diluted in PBS containing 5% BSA and 0.3% Triton X-100. After rinsing three times in PBS with 0.3% Triton X-100 for a total of 45 min, slides were placed for 2 h in secondary antibody. Secondary antibodies (1:500 dilution) included goat anti-mouse Alexa 488 conjugate (Invitrogen, Carlsbad, CA, USA). After rinsing the slides three times in PBS for a total of 45 min, the slides were mounted with Fluoromount-G mounting medium containing Hoechst 33342 stain (Electron Microscopy Sciences, Hatfield, PA, USA). The fluorescent signal was imaged using a Nikon fluorescent microscope and NIS-Elements imaging software and a Photometrics CoolSnap K4 CCD camera.

### Cell Counting

In Experiment 3, cellular quantitation was completed by a blinded reviewer. Three rats were randomly chosen out of the control groups and four rats out of the VZV treated groups and the remaining tissues were frozen fresh for other molecular studies not included in this manuscript. Cell counts were completed on 20 μm coronal sections. The AAV8 injection site was the center point from which sections were selected. Every other section was selected for staining. Typically three sections were counted for each animal. The slides were analyzed using ImageJ software, the average background for the slides within a treatment group was subtracted from the image and a fluorescent signal associated with a cell nucleus was counted as a positive cell.

Counts were completed for the number of AAV8, NeuN or VGLUT2 positive cells within a 0.5 mm^2^ circular field adjacent to the injection site. On each section two randomly selected fields near the tip of the injection site was counted. Counts were completed within the ventral thalamic nuclei including the VMP, VPL and Rt thalamic nuclei (Supplementary Figure S2). Counts were also completed in the ZI (Supplementary Figure S2).

Cell counts from the two fields on each section were then averaged. This average count for the three sections was averaged for each animal. Values were given as a mean and standard error of the mean (SEM) representing an average of the values for the three or four animals in each treatment group.

### Statistics

LFP and cell count data were analyzed by two-way ANOVA, the independent variables were virus (VZV, control) and/or drug (vehicle, CNO) and the dependent variables were the power values and cell counts. Data was analyzed for normality and homogeneity (Prism 5.04, GraphPad Software, La Jolla, CA, USA, or Abstat, Anderson Bell Corp, Arvada, CO, USA). For the PEAP assay the fraction of time each animal spent on the light side of the testing chamber during the 30 min testing period was analyzed using a Generalized Linear Model (GLM) regression with response variable (the time fraction) modeled by the Beta distribution parametrized by the location and precision parameters. This approach allowed us to account for the bounded nature of the response variable from 0.0 to 1.0, while employing the flexibility of the GLM. The precision parameter of the beta distribution was explicitly modeled in addition to the location parameter because the variance of the responses was expected to change with change in the mean response (e.g., the variance should diminish as mean response gets close to either 0.0 or 1.0 boundary). Link function for the location parameter was *loglog* and for the precision parameter was *log*. The responses were modified by applying the following transformation (*y* (*n* − 1) + 0.5)/*n*, where *y* is a response and *n* is the total number of observations (=54). This “shrunk” the range of responses to the open (0.0 1.0) interval required by the model (Smithson and Verkuilen, 2006). Analysis was performed with “betareg” software package (Cribari-Neto and Zeileis, 2010) for R software (R Core Team, 2017). Type of pain induction (control or VZV, “PAIN” term in the models specification), and DREADD activation (vehicle or CNO, “MANIPULATION” term in the model specification)—the independent variables—were coded as binary values. Submodel for location parameter of the beta distribution of responses was specified through a combination of main effects of pain induction and DREADD manipulation, and their interaction (formula = responses ~ PAIN + MANIPULATION + PAIN:MANIPULATION|1). Additional parameters supplied to the “betareg” function included bias correction and numeric computation of the Hessian matrix. To test for the significance of the entire model, it was compared to the intercept-only model for the same response data using Likelihood Ratio test. All values were given as the mean and SEM.

## Results

### Experiment 1—Behavioral Testing of the Affective Pain Response after Trigeminal Ganglion Injection

**Figure 1:**
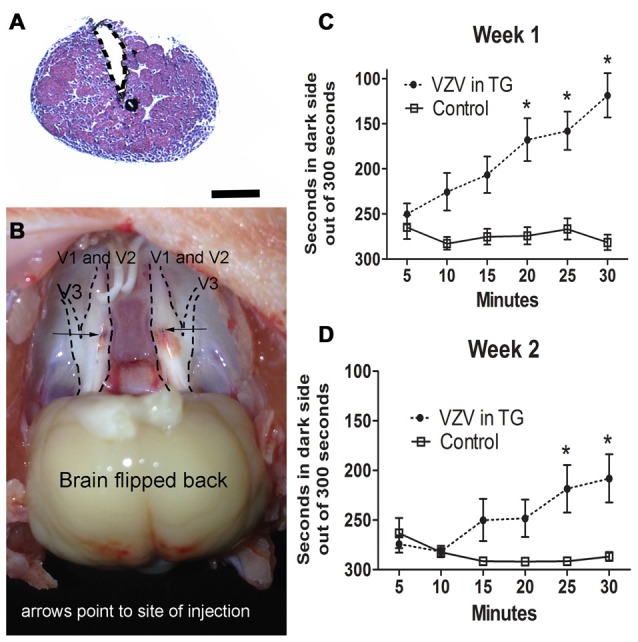
Experiment 1, the affective pain increased after injection of varicella zoster virus (VZV) into the trigeminal ganglion. **(A)** shows a hematoxylin eosin stained coronal section of the trigeminal ganglion after stereotaxic injection of India ink (black). The needle injection site is marked by a dotted line. Bar = 1 mm. In **(B)** a gross dissection of a rat head is shown with the trigeminal ganglion exposed after bilateral stereotaxic injection with India ink (arrows point to the black India ink spots). V1, V2 and V3 refer to the different branches of the trigeminal ganglion. Place escape avoidance paradigm (PEAP) behavioral measurements were completed weekly, **(C)** is 1 week after VZV was injected into the trigeminal ganglion and **(D)** is the second week after VZV was injected into the trigeminal ganglion. Asterisk indicates a significant difference of *p* < 0.05. The values are the mean ± standard error of the mean (SEM). There were 11 animals in the control group and 17 animals in the VZV treatment group.

Injection of the V1/V2 region of the trigeminal ganglion (Figures 1A,B) resulted in affective pain in the orofacial region beneath the eye and just posterior to the whisker pad. The response was significant in week 1 (*F*_(1,156)_ = 60.6, *p* < 0.0001, Figure 1C) and week 2 (*F*_(1,156)_ = 14.9, *p* < 0.001, Figure 1D). Further testing of other regions of the face was not completed.

### Experiment 2—Behavioral Testing after VZV Whisker Pad Injection

**Figure 2:**
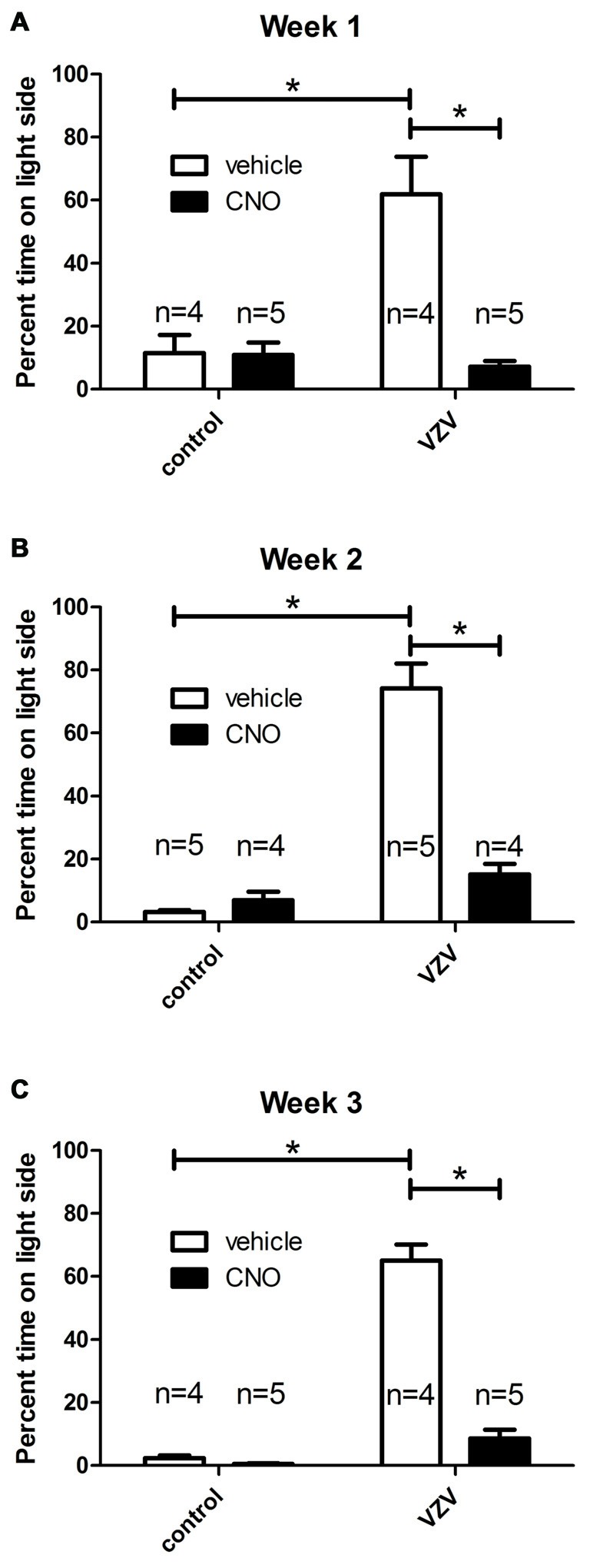
Experiment 2, the orofacial affective pain was elevated after injecting the left whisker pad with VZV. Graphs show the percentage of time spent on the light side of the PEAP testing chamber compared to the dark side of chamber by week. A greater percent of time spent on the light side indicated a greater affective pain. The right ventral posterior medial/posterior lateral thalamus (VPM/VPL) was infused with of adeno-associated virus (AAV8)-hSyn-hM4D(Gi)-mCherry and 1 week later the left whisker pad was injected with VZV or the control (MeWo cells without virus). Thirty minutes before PEAP testing the rats were given an injection of either clozapine-n-oxide (CNO) or vehicle (0.9% saline). Testing was completed once a week for 30 min for three consecutive weeks **(A–C)**. The number of rats in each group is shown on the graphs. The animals receiving CNO or saline were reversed from week to week. Asterisk indicates a significant difference of *p* < 0.05. The values are the mean ± SEM.

**Figure 3:**
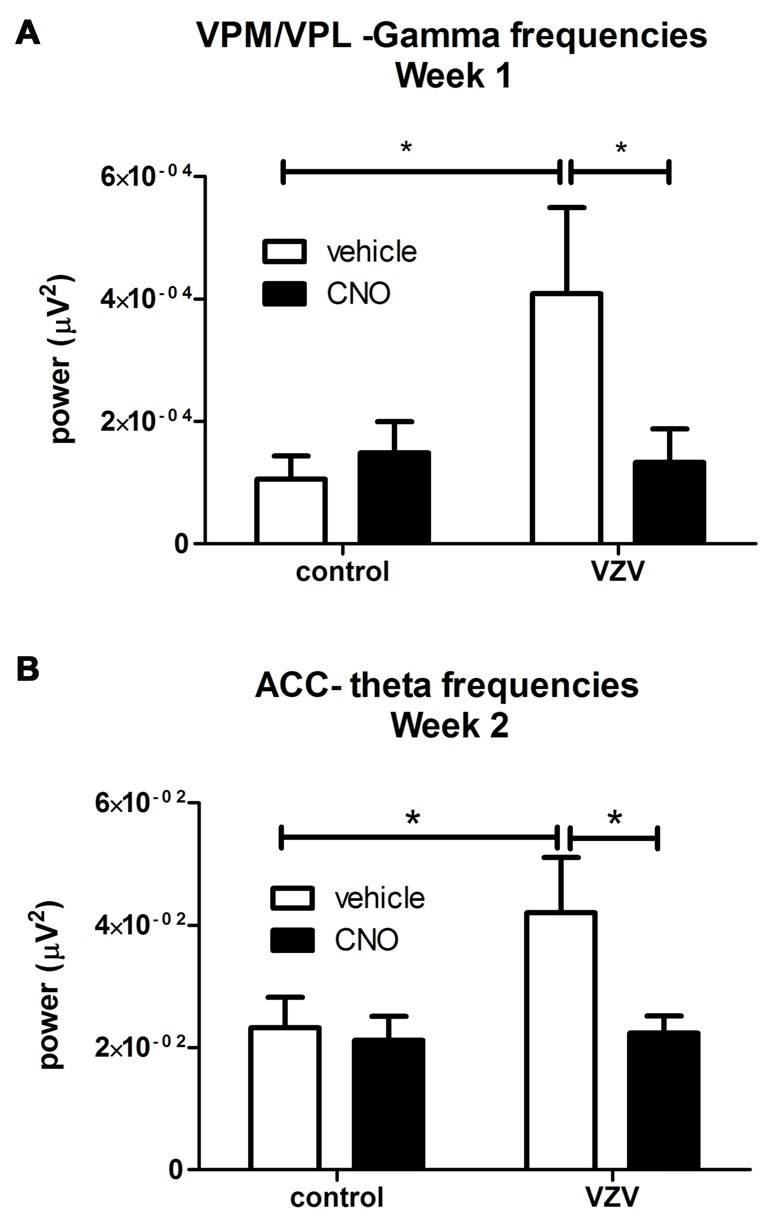
Experiment 3, power spectrum analysis following infusion of the VPM/VPL thalamus with AAV8-hSyn-hM4D(Gi)-mCherry and placement of an electrode in the anterior cingulate cortex (ACC) and VPM/VPL VZV was injected into the whisker pad 1 week after surgery. Local field potential (LFP) recordings were completed in the VPM/VPL **(A)** and ACC **(B)**. Thirty minutes before LFP recording the rats were given an injection of either CNO or vehicle (0.9% saline). Testing was completed once a week for 30 min. Testing was performed on three consecutive weeks. Data for week 1 is shown in **(A)** and the data for week 2 is shown in **(B)**; data for week 3 was not significant and is not shown. Asterisk indicates a significant difference of *p* < 0.05. There were seven animals in each treatment group. The values are the mean ± SEM.

A significant VZV effect was measured 1 (Figure 2A), 2 (Figure 2B) and 3 (Figure 2C), weeks after injection into the whisker pad. Affective aspects of pain are captured by the behavioral assay utilized in these studies (LaBuda and Fuchs, 2000). Affective pain involves central pathways, but infection of the peripheral nerve terminals of the face were not a requirement for induction of a affective pain behavior as direct infusion of VZV into the trigeminal ganglion significantly increased the response (Figure 1).

Infusion of AAV8 resulted in mCherry expression in the VPM/VPL (Figure 5A). The virally infused construct expressed an engineered acetylcholine Gi receptor that upon activation with CNO will act as a neuronal silencer. CNO administration significantly decreased the behavioral response in weeks 1 (Figure 2A), 2 (Figure 2B) and 3 (Figure 2C) consistent with previous results (Kramer et al., 2017).

**Figure 4:**
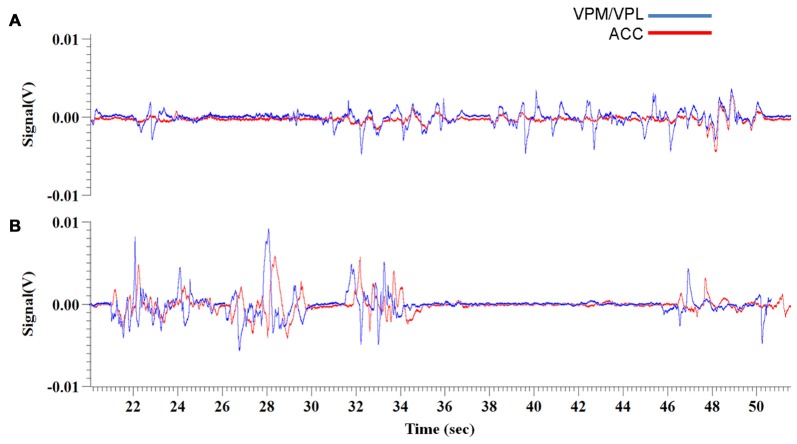
Experiment 3, representative raw traces of LFP recordings from the VPM/VPL thalamus and ACC 1 week and 2 weeks post-VZV injection (**A,B**, respectively). The representative traces show recordings from 20 min to 50 min of the 70 min total recording time.

**Figure 5:**
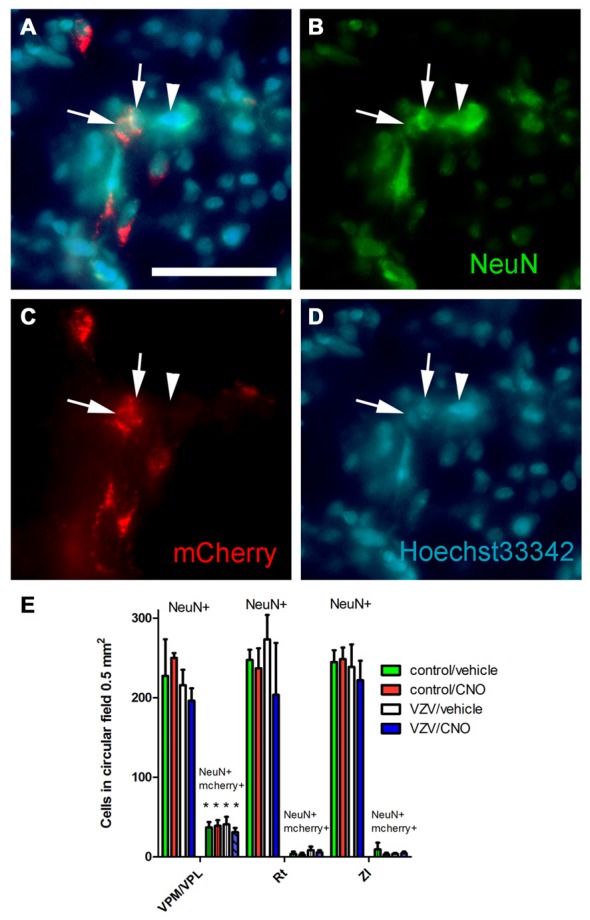
Experiment 3, **(A–D)** immunofluorescent staining of the VPM/VPL thalamus of a rat 4 weeks after infusion of AAV8-hSyn-hM4D(Gi)-mCherry. The representative brain slice was collected from a rat whose whisker pad was injected with control MeWo cells and given an IP injection of vehicle (0.5% saline). Arrows point to NeuN positive cells expressing mCherry, arrowhead points to NeuN positive cell that did not express mCherry. Nuclei were stained blue with Hoechst 33342. Bar equals 50 micrometers. **(E)** Histogram of blinded cell counts in circular field 0.5 mm^2^ in area, counts were completed in randomly chosen fields adjacent to the injection site in several thalamic regions; the VPM, VPL and reticular (Rt) thalamic nuclei, as well as the zona incerta (ZI). Asterisk indicates *p* < 0.05 when comparing the NeuN+/mCherry positive cells in the VPM/VPL to the NeuN+/mCherry positive cells in the Rt and ZI. *n* = 3 for the control/vehicle and control/CNO groups and *n* = 4 for the VZV/vehicle and VZV/CNO groups. Values are the mean and SEM.

The response to CNO was not significant in the absence of DREADD (Supplementary Figure S3).

Parameter estimates, their standard errors, z test statistic values and associated *p*-values for the location parameter submodel are presented in Table 2. In the location parameter submodel (i.e., the mean response), the intercept, the main effect of pain induction type (control vs. VZV), and pain induction—DREADD manipulation interaction effect (that is, the effect of DREADD manipulation being dependent on the level of the pain induction) were highly significant. The effect of DREADD manipulation by itself was not significant. The likelihood ratio test against an intercept-only model yielded a chi-square value of 81.311 (*df* = 3) and a *p*-value < 2.2E-16.

**Table 2:** Parameter estimates, their standard errors, *z* test statistic values and associated *p*-values for the location parameter in the beta generalized linear model (GLM) model for the time fraction place escape avoidance paradigm (PEAP) data.

	Estimate	Std. Error	*z* value	Pr(>|z|)	Signif.
(Intercept)	−0.94455	0.08973	−10.526	<2e-16	***
Pain	1.91662	0.1672	11.463	<2e-16	***
Manipulation	−0.01441	0.11745	−0.123	0.902	
Pain:Manipulation	−1.70977	0.20315	−8.416	<2e-16	***

Parameter estimate, its standard error, *z* test statistic value and associated *p*-value for the precision parameter submodel are presented in Table 3. For precision parameter submodel, the Intercept parameter was highly significant. Pseudo R-squared coefficient (squared correlation coefficient between the linear predictor and link-transformed observations) was 0.7895.

**Table 3:** Parameter estimate, its standard error, *z* test statistic value and associated *p*-value for the precision parameter in the beta GLM model for the time fraction PEAP data.

	Estimate	Std. Error	*z* value	Pr(>|z|)	Signif.
(Intercept)	2.4484	0.1996	12.27	<2e-16	***

### Experiment 3—Field Potentials in VPM/VPL of Awake Animals

VZV injection significantly increased LFP activity at the theta frequencies in the VPM/VPL the first week after injection (*F*_(1,27)_ = 3.89, *p* = 0.05, *n* = 7). VZV also increased VPM/VPL activity at the gamma frequencies in week 1 (*F*_(1,27)_ = 4.18, *p* = 0.05). A significant interaction between virus and drug (CNO) treatment was observed in week 1 (*F*_(1,27)_ = 5.10, *p* = 0.03). *Post hoc* analysis demonstrated VZV significantly increased gamma frequency activity and CNO treatment decreased this response (Figure 3A). No significant difference was observed between the groups before CNO/saline injection for any wavelength. Moreover, no significant change in LFP activity was observed in weeks 2 and 3.

### Experiment 3—Field Potentials in ACC of Awake Animals

No significant effect was observed in week 1 after VZV injection, but in week 2 VZV significantly increased activity in the theta frequencies (*F*_(1,27)_ = 3.7, *p* = 0.05, *n* = 7). An interaction between virus and drug was not observed (*F*_(1,27)_ = 2.86, *p* = 0.10) in the ACC during week 2. *Post hoc* analysis indicated VZV treatment significantly increased theta activity and CNO treatment prevented this increased activity (Figure 3B). No significant effect was observed in the ACC during weeks 1 and 3.

### Experiment 3—Coherence of ACC and VPM/VPL Field Potentials

Coherence is the phase relationship between two separate signals, 0 indicates no coherence, or no phase relationship while 1 indicates a strong coherence, or a constant phase relationship. Coherence was investigated by using Spike2 software to create a waveform correlation comparing the raw electrode data trace between the VPM/VPL and ACC for each field potential recording. Significance between conditions at each week was then determined using a *t*-test to compare the means.

For the first week of testing, the mean correlation between VPM/VPL and ACC waveforms was 0.58 (Figure 4A). There was not a significant change in coherence between the control condition (i.e., before use of Von Frey filaments) (0.59 ± 0.065) and when the animals were being exposed to the Von Frey filaments during the PEAP test (0.58 ± 0.05). In the second week of testing the mean correlation between the two waveforms was 0.74 (Figure 4B). In this week there was a significant difference (*p* < 0.001) in coherence, with the control condition having a higher level of waveform coherence (0.85 ± 0.03) vs. the period of Von Frey testing (0.64 ± 0.05). Note the strong coherence between the VPM/VPL and ACC waveforms in the control condition during the second week. In the final week the coherence between the raw trace waveforms was 0.75. In this week there was also a significant difference (*p* = 0.003) in the coherence of the ACC and VPM with the control condition (0.83 ± 0.03) once again being more correlated than during Von Frey testing (0.67 ± 0.03).

### Experiment 3—Excitatory Neurons in the VPM/VPL Were Transduced with Gi Construct

Thalamic neurons were transduced with the Gi construct after injecting the VPM/VPL with AAV as indicated by the mCherry signal (Figure 5A). Following AAV injection the whisker pad of these rats were injected with either VZV or control (i.e., MeWo cells). One week following whisker pad injection the groups were divided, half received the drug CNO and half vehicle. The average number of NeuN positive cells in a 0.5 mm^2^ field was not significantly different between the treatment groups (*F*_(3,31)_ = 0.94, *p* = 0.42, *n* = 3 control groups, *n* = 4 VZV groups) or between the different nuclei (VPM/VPL, Rt or ZI) (*F*_(2,31)_ = 0.32, *p* = 0.72). No significant interaction between treatment and location was observed (*F*_(6,31)_ = 0.25, *p* = 0.95). The Gi construct with a fluorescent tag (mcherry) was observed in cells positive for the neuronal marker NeuN (Figures 5A–D). Cell counts of these fluorescent cells indicated that the VPM/VPL had a significantly greater number of AAV transduced neurons (*F*_(2,31)_ = 36.66, *p* < 0.0001) than the Rt or ZI (Figure 5E). There was no effect of treatment (*F*_(3,31)_ = 0.33, *p* = 0.80) and no interaction between treatment and location of the cells (*F*_(6,31)_ = 0.35, *p* = 0.90).

### Experiment 3—Neuron Staining

**Figure 6:**
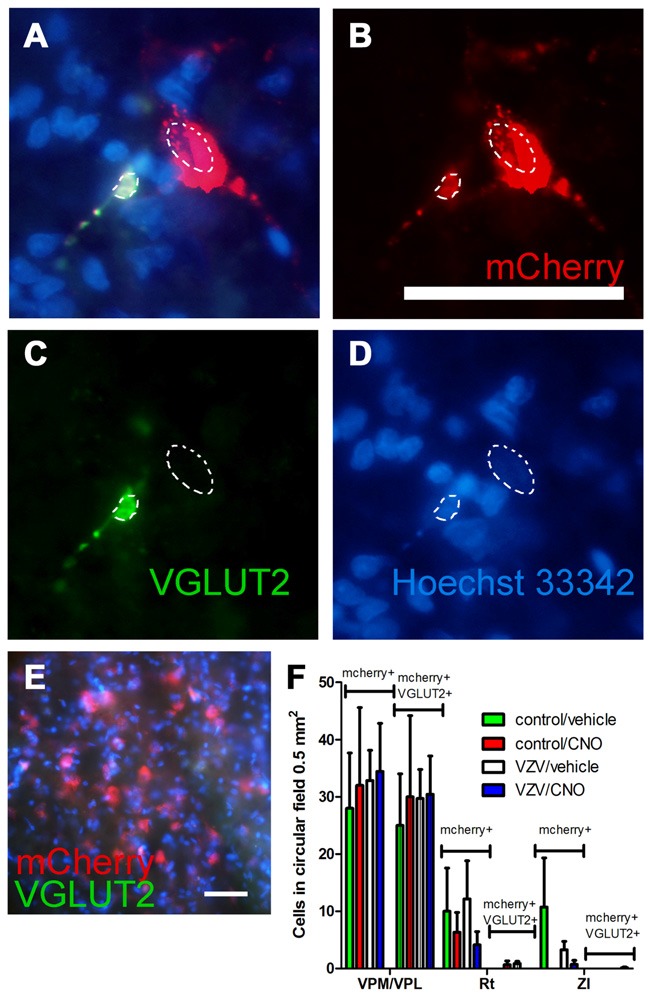
Experiment 3, **(A–D)** immunofluorescent staining of the VPM/VPL thalamus of a rat 4 weeks after infusion of AAV8-hSyn-hM4D(Gi)-mCherry (red). Two nuclei are outlined with a dashed line. Vesicular glutamate transporter 2 (VGLUT2) is a marker of glutamatergic excitatory neurons. Immunostaining of the brain section indicated VGLUT2 (green) and the neuron silencer virus AAV8 (red) were co-expressed in some cells (**A**, yellow). In **(A–D)** the representative animal was injected with control (MeWo) cells and given an IP injection of vehicle (0.5% saline). **(E)** shows a coronal brain slice after staining with secondary antibody but omitting the VGLUT2 antibody. Nuclei were stained blue with Hoechst 33342. Bar equals 50 micrometers. **(F)** AAV8 infected (mCherry+, see bracket) and mCherry+ cells that co-localize with VGLUT2 (mCherry+ and VGLUT2+, see bracket) were counted in circular fields 0.5 mm^2^ in area. Counts were completed in randomly chosen fields adjacent to the injection site in several thalamic regions; the VPM, VPL and reticular (Rt) thalamic nuclei, as well as the ZI. *n* = 3 for the control/vehicle and control/CNO groups and *n* = 4 for the VZV/vehicle and VZV/CNO groups. Values on the histogram are the mean and SEM.

The AAV8 transduced cell population was characterized by staining for the excitatory neuronal marker VGLUT2 (Figures 6A–D, arrows). Omission of the VGLUT2 antibody in the staining protocol resulted in no detectable VGLUT2 signal within the thalamic region (Figure 6E). The number of mCherry and VGLUT2 positive cells was significantly greater in the VPM/VPL vs. the Rt or ZI (*F*_(2,31)_ = 27.56, *p* < 0.0001). Treatment had no effect on the number of AAV transduced cells that stained for VGLUT2 (*F*_(3,31)_ = 0.08, *p* = 0.96) nor was there a significant interaction between treatment and the location of the cells (*F*_(6,31)_ = 0.06, *p* = 0.99). The data show a majority of the mCherry positive cells (i.e., contained the silencer construct) were VGLUT2 positive excitatory neurons (Figure 6F) which is consistent with the behavioral affective pain response and LFP findings being related to a decrease in glutamatergic neuron activity.

## Discussion

Relief of VZV-induced pain can be observed when treating shingles patients with topical aspirin/diethyl ether and lidocaine (Hempenstall et al., 2005). The efficacy of this topical treatment suggests VZV pain involves peripheral neurites. Moreover, neurite retraction within the whisker pad and paw can be observed after VZV injection (Guedon et al., 2015; Stinson et al., 2017), consistent with humans that have VZV-induced neuralgia (Guedon et al., 2015). To address whether VZV-induced pain was due to damage at the peripheral nerve terminals, we injected VZV into the trigeminal ganglion. The strong affective pain response after injecting the trigeminal ganglion suggests that the pain mechanism was not restricted to the terminals. Support for this idea comes from studies by the Kinchington lab demonstrating that expression of at least some viral proteins in the ganglia are necessary to induce the affective pain in animals (Guedon et al., 2014). For example, the VZV immediate early gene IE62 is present in humans with HZ and in VZV infected rat neurons (Kress and Fickenscher, 2001; Schaap-Nutt et al., 2006; Hama et al., 2010; Stinson et al., 2017). Dr. Kinchington (personal communication) has evidence that expression of this viral transcription factor alters neuronal function and that this factor is necessary for the pain response.

The relationship between affective pain and LFP activity in the thalamus and ACC was investigated after injecting VZV into the whisker pad. Because affective aspects of pain were quantitated in this study (i.e., the PEAP test) and the ACC has an important role in the affective pain response we studied activity in the ACC (LaBuda and Fuchs, 2000; Fuchs et al., 2014). VZV injection significantly increased the orofacial affective pain and increased LFP activity in the thalamus and ACC. Activation of Gi in the VPM/VPL through the administration of CNO attenuated the affective pain and reduced LFP in the thalamic region. Interestingly, LFP activity in the ACC also diminished consistent with a functional VPM/VPL-ACC connection as previously reported (Wang et al., 2007). Interestingly, the Wang et al. study used a heat pain and our study induced pain by injecting virus suggesting that the ACC-thalamic functional connection does not necessarily depend on the modality by which pain was induced (Wang et al., 2007).

Inflammatory and visceral pain has been associated with changes in the low frequency band of the ACC, consistent with our observation that the theta frequency band significantly increased upon VZV treatment (Wang J. et al., 2015; Harris-Bozer and Peng, 2016). These studies are the first to demonstrate an increase in the gamma frequency band within the thalamus of a rat after VZV-induced pain. An increase in gamma frequency oscillation has been measured in the somatosensory cortex of humans during an induced pain condition (Gross et al., 2007; Lau et al., 2012). Moreover, coherence in the thalamus and ACC was affected by treatment which may indicate a mechanistic role in the chronic pain response (Wang et al., 2007; Wang J. et al., 2015). The changes in specific frequencies are expected to provide insight into the processing of the sensory information and the pain response.

Although less than 30% of the neurons adjacent to the recording electrode in the VPM/VPL were AAV infected, inhibition of the LFP was nearly complete after administration of CNO. The robust LFP response would be expected if the region near the tip of the electrode had nearly complete AAV infection resulting in expression of CNO controlled Gi activation. Our data indicated less than 30% of the cells were AAV infected. The cell counts completed in this study included the region very near the recording electrode tip but counts did include regions extending from the recording electrode tip and would potentially include regions of reduced infection. We did not count the percentage of infected cells vs. distance from the injection site and have not tested this idea directly but it is possible that the method of the cell counting resulted in a lower than expected infection rate. Alternatively, if electrically active cells preferentially became infected with AAV this would result in a low percentage of infectivity but a highly effective suppression of the LFP.

Approximately two-thirds of the virus infected cells contained the excitatory glutamatergic marker VGLUT2. Glutamate is a key signaling molecule in the thalamic region, relaying pain from periphery (Bhave et al., 2001; Osikowicz et al., 2013; Acher and Goudet, 2015). Lower levels of glutamate have been associated with analgesia (Abarca et al., 2000; Naderi et al., 2014) and increased levels of glutamate in the thalamus are associated with various pain conditions (Salt and Binns, 2000; Silva et al., 2001; Likavcanová et al., 2008; Ghanbari et al., 2014; Salt et al., 2014; Wang Z. T. et al., 2015; Zunhammer et al., 2016). Based on the DREADD results and the known effect of glutamate in the thalamus, activation of Gi will likely lead to inhibition of the glutamatergic neuronal population in the thalamus that would then attenuate the affective pain response.

This is the second study to look at the role of specific brain regions in VZV-induced pain. In the first study GABA neurons in the reticular thalamic region were shown to modulate VZV pain (Stinson et al., 2017). In the current work we demonstrate for the first time a role for excitatory glutamatergic neurons in the VMP/VPL. We also have identified for the first time the effect of VZV on electrical activity, both in the VMP/VPL and ACC. Previous work in other laboratories demonstrated that mechanical allodynia and thermal hyperalgesia are induced by VZV injection of the paw (Fleetwood-Walker et al., 1999; Dalziel et al., 2004; Garry et al., 2005; Kinchington and Goins, 2011). In addition previous work demonstrated that spinal astrocytes have a role in the sensory pain response because inhibition of astrocytes in the spine reduced VZV hypersensitivity (Zhang et al., 2011). Thus, in addition to studying the brain function in VZV pain we have demonstrated the role of these brain regions and the associated glutamatergic cells in the affective pain response.

In conclusion, we have a rat model that allows study of VZV disease mechanisms resulting in pain within the orofacial region. Our results suggest the mechanism for VZV affective pain is not restricted to the peripheral nerve terminals, but involves thalamic excitatory neurons. Importantly, attenuation of excitatory neuronal activity in the thalamus resulted in an attenuation of electrical activity in the ACC, which was associated with a reduction in affective pain. This association is consistent with the idea that excitatory neurons within the VPM/VPL modulate effective aspects of VZV-induced affective pain by altering activity in the ACC.

## Author Contributions

PRKramer, PRKinchington, YBP, JS and LLB contributed in the design of the experiments and analysis of the data as well as contributing to the drafting and editing of the manuscript. JS, CS and MBY collected data, performed experiments and analyzed the data for this study. MU performed data analysis and contributed to writing the manuscript.

## Conflict of Interest Statement

The authors declare that the research was conducted in the absence of any commercial or financial relationships that could be construed as a potential conflict of interest. The reviewer VG-S and handling Editor declared their shared affiliation.
