# Supplementary material for: Role for the Ventral Posterior Medial/Posterior Lateral Thalamus and Anterior Cingulate Cortex in Affective/Motivation Pain Induced by Varicella Zoster Virus
Source: Front Integr Neurosci. 2017 Oct 16;11:27. doi: 10.3389/fnint.2017.00027 (PMC5651084; doi:10.3389/fnint.2017.00027)

Supplemental Figure 1. Spontaneous facial grooming before and after whisker pad injection. Front and hind paw rubbing, as well as, facial rubbing on the floor were measured in seconds out of a 600 second measurement period. Fourteen animals were in the control group and fifteen animals were in the VZV group. A measurement was taken before injection and one week after whisker pad injection of either 0.9% saline (Control) or 650,000 pfu/whisker pad of VZV. No thalamic injections were completed in this experiment.

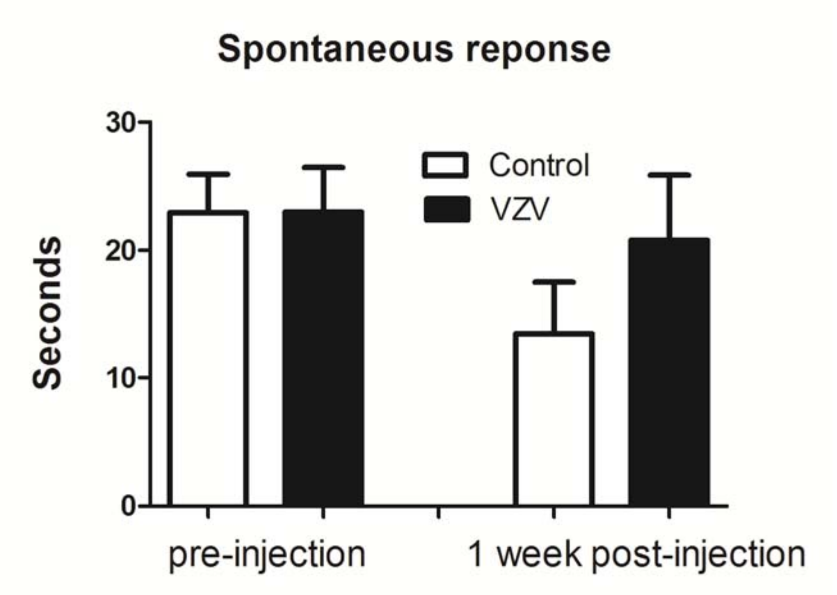

Supplement: Supplementary file 1 [file Image_1.pdf]
