# Supplementary material for: Role for the Ventral Posterior Medial/Posterior Lateral Thalamus and Anterior Cingulate Cortex in Affective/Motivation Pain Induced by Varicella Zoster Virus
Source: Front Integr Neurosci. 2017 Oct 16;11:27. doi: 10.3389/fnint.2017.00027 (PMC5651084; doi:10.3389/fnint.2017.00027)

Supplemental Figure 2. A composite image of a Nissl stained section 3.6 mm posterior to Bregma shows the regions that were used in counting cells. Po= posterior thalamic nuclei, VPM= ventral posteromedial thalamic nuclei, VPL= ventral posterolateral thalamic nuclei, ZI= zona incerta, IC= internal capsule and Rt= reticular thalamic nuclei, LV= lateral ventricle (arrow).

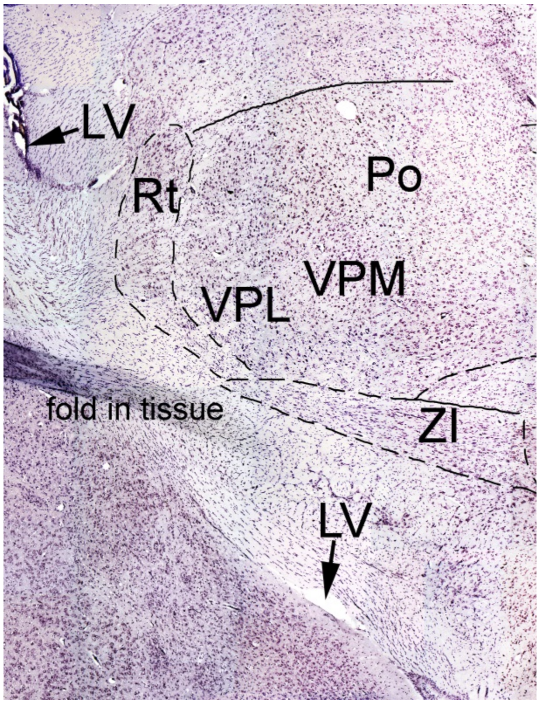

Supplement: Supplementary file 2 [file Image_2.pdf]
