# Supplementary material for: Role for the Ventral Posterior Medial/Posterior Lateral Thalamus and Anterior Cingulate Cortex in Affective/Motivation Pain Induced by Varicella Zoster Virus
Source: Front Integr Neurosci. 2017 Oct 16;11:27. doi: 10.3389/fnint.2017.00027 (PMC5651084; doi:10.3389/fnint.2017.00027)

Supplemental Figure 3. Clozapine-n-oxide injection did not alter animal movement during the place escape avoidance paradigm in rats without DREADDs. The thalamus of rats were infused with AAV8 virus expressing a neuronal silencing construct hSyn-hM4D(Gi)-mCherry (+ DREADD) or 350 mM NaCl, 5% sorbitol in PBS (no DREADD). Then all the rats whisker pads were injected with 100,000 pfu of VZV a week after thalamic infusion. One week later PEAP testing was initiated. CNO or vehicle (0.9% saline) was injected IP 30 minutes before each round of PEAP testing. PEAP testing was completed weekly for three weeks. There were 4-5 rats per treatment group. A green asterisk indicates a significant difference between the vehicle/+ DREADD group and the CNO/+ DREADD group. A red asterisk indicates a significant difference between the CNO/no DREADD group and the CNO/+ DREADD group. Statistics included two-way ANOVA and Bonferroni post-hoc tests.

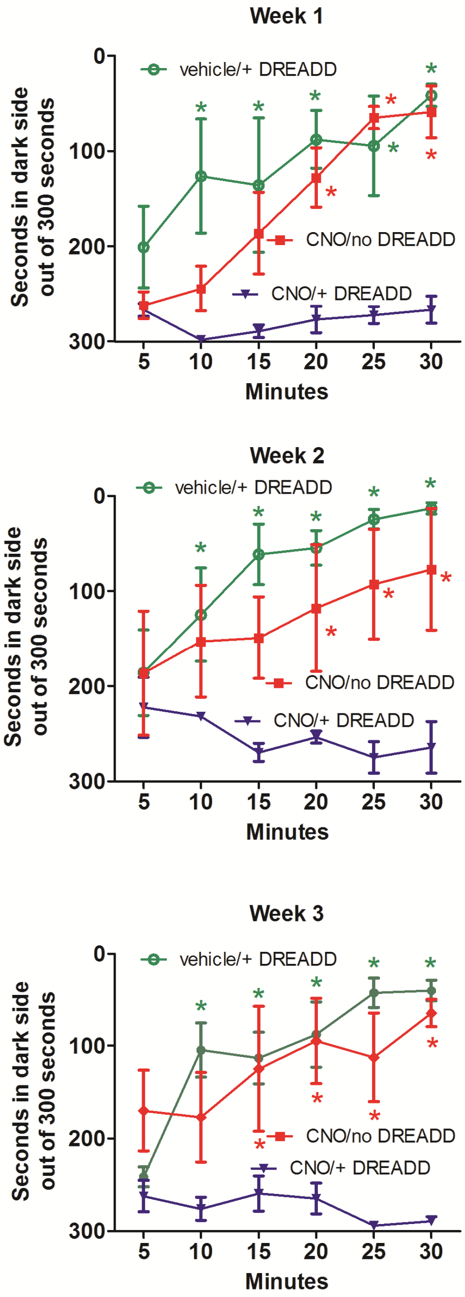

Supplement: Supplementary file 3 [file Image_3.pdf]
